# Crystallization of SHARPIN using an automated two-dimensional grid screen for optimization

**DOI:** 10.1107/S1744309112022208

**Published:** 2012-06-28

**Authors:** Benjamin Stieglitz, Katrin Rittinger, Lesley F. Haire

**Affiliations:** a Division of Molecular Structure, MRC – National Institute for Medical Research, The Ridgeway, Mill Hill, London NW7 1AA, England

**Keywords:** SHARPIN, seeding

## Abstract

An N-terminal fragment of human SHARPIN was recombinantly expressed in *Escherichia coli*, purified and crystallized. Crystals suitable for X-ray diffraction were obtained by a one-step optimization of seed dilution and protein concentration using a two-dimensional grid screen. The crystals belonged to the primitive tetragonal space group *P*4_3_2_1_2, with unit-cell parameters *a* = *b* = 61.55, *c* = 222.81 Å. Complete data sets were collected from native and selenomethionine-substituted protein crystals at 100 K to 2.6 and 2.0 Å resolution, respectively.

## Abstract 2

The expression, purification and crystallization of an N-terminal fragment of SHARPIN are reported. Diffraction-quality crystals were obtained using a two-dimensional grid-screen seeding technique.

## 1. Introduction

SHARPIN (SHANK-associated RH-domain interacting protein) is a cytosolic 45 kDa protein that was originally identified as a scaffolding partner for SHANK proteins in the postsynaptic density of excitatory synapses (Lim *et al.*, 2001 ▶). Recently, it has been shown that SHARPIN also plays an important role in immunity and inflammation by stimulating the formation of linear ubiquitin chains. SHARPIN is a constituent of the linear ubiquitin chain-assembly complex (LUBAC) and the absence of SHARPIN causes dysregulation of NF-κB and apoptotic signalling pathways (Ikeda *et al.*, 2011 ▶; Gerlach *et al.*, 2011 ▶; Tokunaga *et al.*, 2011 ▶). The C-terminal half of SHARPIN consists of an ubiquitin-like (Ubl) domain followed by an Npl4-zinc finger (NZF) domain and is important for complex formation with the LUBAC component HOIP (haem-oxidized iron-regulatory protein 2 ubiquitin ligase-1 interacting protein) and with ubiquitin (Ikeda *et al.*, 2011 ▶; Gerlach *et al.*, 2011 ▶; Tokunaga *et al.*, 2011 ▶). The N-terminus of SHARPIN has been reported to mediate homomultimerization (Lim *et al.*, 2001 ▶). However, the structural basis of SHARPIN self-association has not been investigated. To obtain insight into the oligomeric state of SHARPIN, we crystallized residues 1–127 encompassing the region responsible for self-association.

## 2. Experimental

### 2.1. Overexpression and purification

Human SHARPIN 1–127 was cloned into pGEX-4T1 (GE Healthcare) by PCR with the primers Spn1-*Bam*HI (CGCGGATCCATGGCGCCGCCAGCG) and Spn127-*Eco*RI (GCGGAATTCCTAGCTGCCATTCTGTCC). Because this fragment of SHARPIN only contained the N-terminal methionine, two additional methionines were introduced at positions 22 and 101 for selenomethionine (SeMet) derivatization. The L22M/L101M mutant was generated by site-directed mutagenesis with the primers Spn-L22M (GCCGCA­GTGCTCATGGCTGTGCACG) and Spn-L101M (CCTGGAACC­CTCAGCATGCACTTCCTCAACC) according to the QuikChange protocol (Stratagene) and was verified by DNA sequencing. Wild-type and mutant constructs were expressed as GST-fusion proteins using *Escherichia coli* BL21 as a bacterial expression strain in Luria–Bertani broth or SeMet-substituted medium (Molecular Dimensions Ltd) containing 100 µg ml^−1^ ampicillin. The purification procedure was the same for the wild-type and SeMet proteins. Cells were grown to an OD_600_ of 1.2 at 310 K and were induced with 0.1 m*M* isopropyl β-d-1-thiogalactopyranoside (IPTG). After 16 h expression at 298 K, the cell pellets were resuspended in buffer *A* (100 m*M* HEPES pH 7.4, 500 m*M* NaCl, 1 m*M* DTT) and lysed by sonication. The lysate was cleared by centrifugation and loaded onto a column containing 25 ml Glutathione Sepharose Fast Flow medium (GE Healthcare). The immobilized fusion protein was extensively washed with buffer *A* before the GST tag was cleaved overnight with five units of thrombin per milligram of fusion protein. The cleaved protein was further purified by gel filtration on a Superdex S75 column (GE Healthcare) with buffer *B* (50 m*M* HEPES pH 7.4, 150 m*M* NaCl, 1 m*M* DTT) as the elution buffer. 1 l medium typically yielded ∼12 mg purified SHARPIN. The protein was concentrated to a final concentration of 60 mg ml^−1^, flash-cooled in liquid nitrogen and stored at 193 K.

### 2.2. Crystallization

Screening experiments were carried out in 96-reservoir two-well plates (Swissci, Molecular Dimensions Ltd) using an Oryx8 crystallization robot (Douglas Instruments Ltd). Sitting drops were prepared by mixing 100 nl protein solution at 10 mg ml^−1^ (diluted with buffer *B*) with an equal volume of screen solution and equilibrating against 75 µl reservoir solution at 291 K. The first screen used was a simple systematic soluble protein crystallization screen. This was a modification of the ‘Imperial College Screen’ (Haire, 1999 ▶), which sampled a range of precipitants, varying both concentration and pH, to assess the solubility behaviour of the protein under various conditions. Ammonium sulfate and polyethylene glycol (PEG) 3350, representing the most commonly used precipitants, were screened in the ranges 1.0–2.3 *M* and 5–25%(*w*/*v*), respectively, with 0.1 *M* sodium acetate pH 4.6, 0.1 *M* MES pH 6.0, 0.1 *M* PIPES pH 6.8, 0.1 *M* HEPES pH 7.5 or 0.1 *M* Tris–HCl pH 8.3. PEG 3350 at 20%(*w*/*v*) was also screened with either 0.2 *M* ammonium acetate or lithium sulfate at the same pH values. Other precipitants in this screen included sodium potassium phosphate pH 7.5 at 1.4–2.4 *M*, PEG 400 at 25–40%(*v*/*v*), 2-methyl-2,4-pentanediol at 10–50%(*v*/*v*) and propan-2-ol at 5–40%(*v*/*v*), all with 0.1 *M* HEPES pH 7.5. The original screen was expanded to 96 conditions by the addition of triammonium citrate conditions at concentrations varying from 1 to 1.6 *M* at pH 5.5, 6.5, 7.5 and 8.5.

The plate was imaged using an RI54 imager (Formulatrix, USA) immediately after setup and the drops were evaluated by their appearance: whether they remained clear, precipitated or showed phase separation. In this screen, salts showed the most promising trends, with clear drops progressing to white precipitate. PEG gave denatured brown precipitate under most conditions. On the basis of these observations, a sparse-matrix salt screen (72 conditions) was selected for further screening. This was designed by selecting a range of salt conditions from commercially available sparse-matrix screens and reformatting them into a new salt screen. It contained the anions sulfate, phosphate, citrate, tartrate, formate, acetate and malonate, and the cations ammonium, sodium, potassium, lithium and magnesium, with a range of different buffers from pH 4.2 to 9.55.

**Figure 1:**
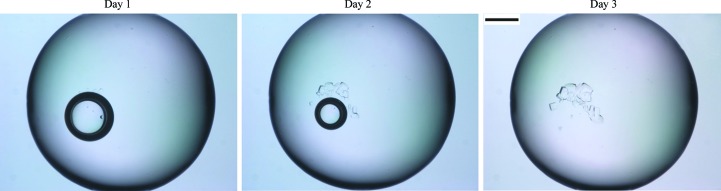
Time course of crystal growth from day 1 to day 3. The scale bar represents 0.2 mm.

The only crystals obtained with this screen were from 4 *M* sodium formate (no added buffer), where an air bubble was initially present in the drop (Fig. 1 ▶). After 12 h, crystals could be observed nucleating near the bubble. These crystals grew and after 3 d the bubble had disappeared. Another drop set up at the same time with 4 *M* sodium formate but in a different screen remained clear, indicating that the bubble may have facilitated nucleation. A crystal was X-rayed to confirm that it was protein and the remaining crystals in the drop were then used for preparation of a microseed solution. No crystals were obtained from any of the other initial screens.

Traditionally, refinement of crystallization conditions to optimize crystal size and quality is carried out using a strategy such as the ‘grid screen’ described by Cox & Weber (1988 ▶). This approach involves successive automated vapour-diffusion experiments in which the precipitant concentration and solution pH are varied in a systematic fashion from an initial coarse grid screen to finer grids. The time required to prepare the appropriate reservoir solutions for the series of grid screens is a drawback of this method. The novel optimization technique described here is a one-step procedure that requires the preparation of only one solution for all of the reservoirs of the crystallization plate, resulting in a considerable time saving. In this two-dimensional grid screen, variation of both protein concentration and seed dilution in the crystallization droplet is used for refinement of growth conditions, rather than variation of the chemical components (Cox & Weber, 1988 ▶). An increase in drop volume from 0.2 to 0.6 µl facilitates crystal removal from the drop and allows larger crystals to grow over time. This method has proved successful in the reproducible growth of diffracting crystals for SHARPIN.

**Figure 2:**
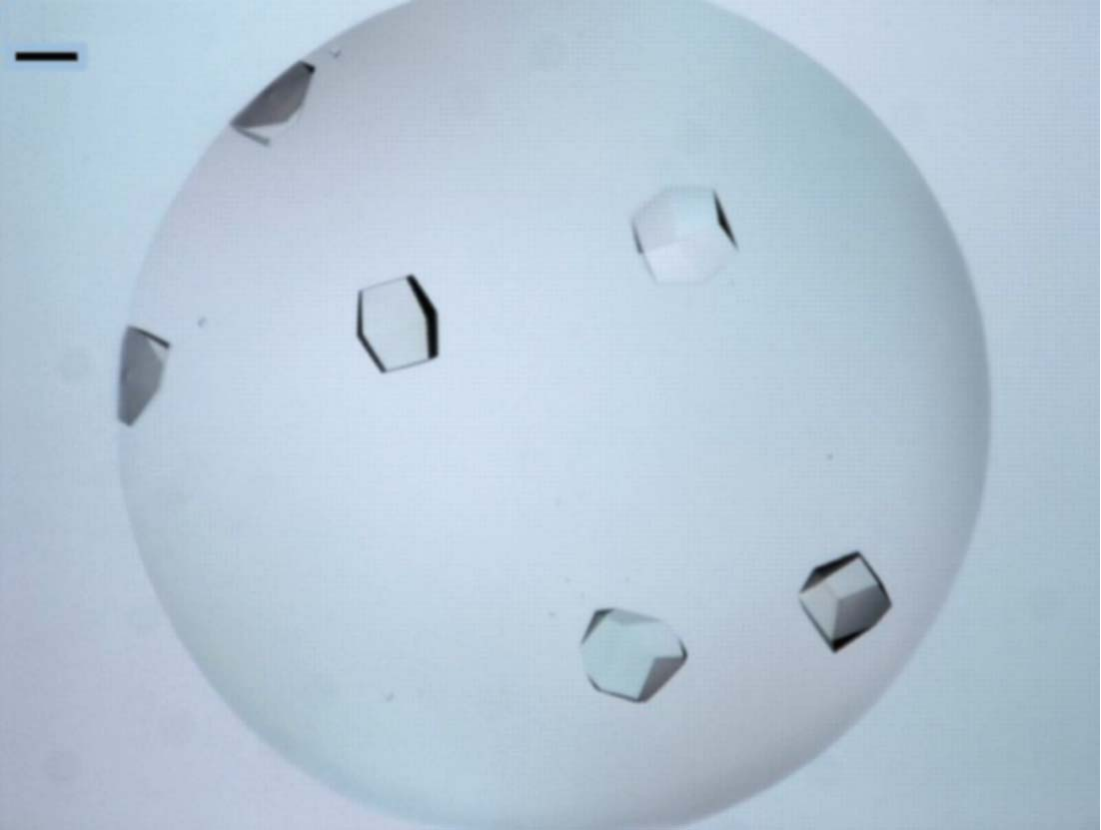
Typical crystals of native SHARPIN obtained using the two-dimensional grid seeding method with 4 *M* sodium formate as precipitant. The crystallization drop (0.6 µl) contained 3.9 mg ml^−1^ SHARPIN (final concentration) with the addition of 45 nl native seed solution in 4 *M* sodium formate. The scale bar represents 0.1 mm.

The two-dimensional grid microseeding screen (Haire, 2011 ▶) uses a script (Douglas Instruments Ltd) where two variables may be varied simultaneously, *e.g.* protein concentration across the plate (*X*) and additive concentration or seed stock up and down the plate (*Y*). Droplets consisted of a total volume of 0.6 µl. The protein concentration in the drop was varied from 2.5 to 5 mg ml^−1^ (in eight increments of 0.35 mg ml^−1^) by addition of buffer *B* as a diluent along the *X* axis of the Swissci plate. The volume of seed solution varied from 0 to 100 nl (in 12 increments of 9 nl) by addition of 4 *M* sodium formate as a diluent along the *Y* axis. All reservoirs contained 75 µl 4 *M* sodium formate. Plates were sealed with clear tape from Hampton Research and incubated at 291 K in the RI54 imager. Images were collected every 12 h for the first 4 d (Fig. 2 ▶).

Native protein and seeds were used in a two-dimensional grid screen to obtain native SHARPIN crystals. The seed-stock solution was prepared by crushing the crystals with a microtool (Hampton Research, USA) and transferring them into an Eppendorf tube containing a bead (using the Seed Bead kit from Hampton Research) with 50 µl 4 *M* sodium formate as a stabilizing solution. The crystals were then mechanically homogenized on a standard laboratory vortex mixer for 3 min at full speed (D’Arcy *et al.*, 2007 ▶).

**Figure 3:**
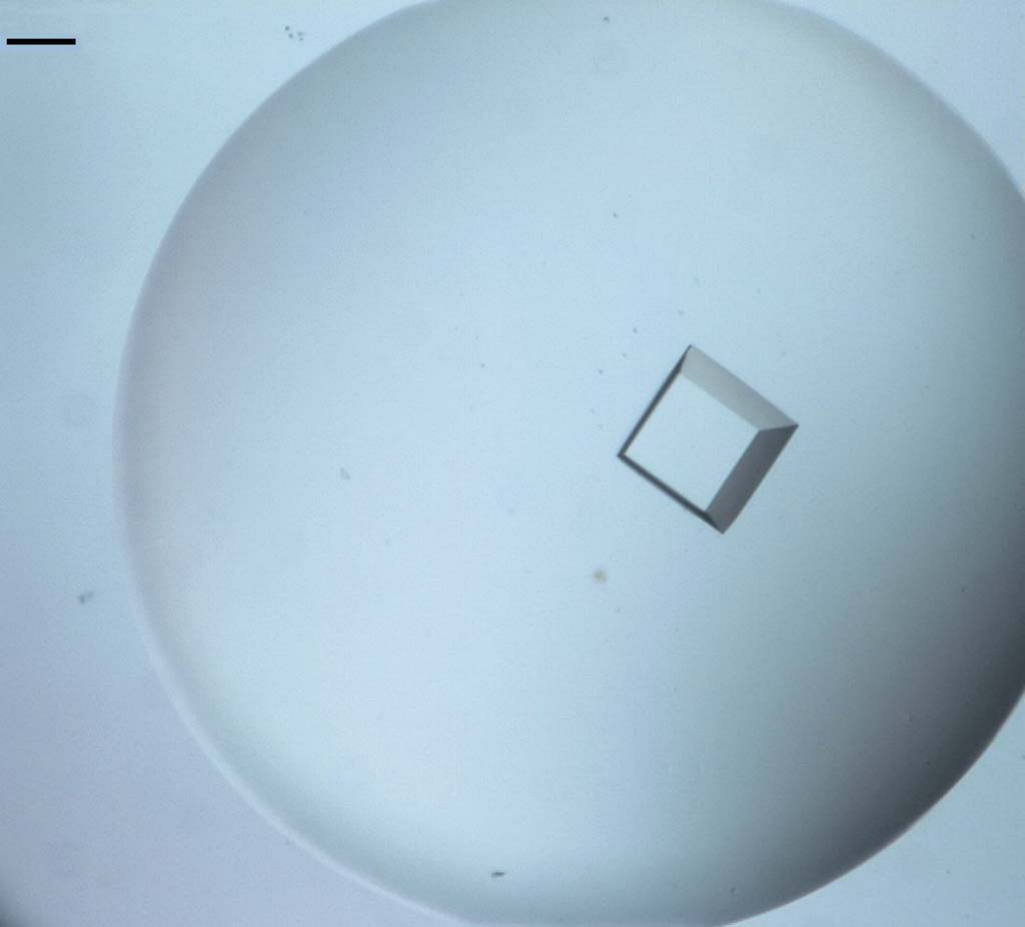
A typical crystal of SeMet SHARPIN grown by cross-seeding with native seeds in a two-dimensional grid experiment. The crystallization drop (0.6 µl) contained 3.9 mg ml^−1^ SeMet SHARPIN (final concentration) with the addition of 9 nl native seed solution in 4 *M* sodium formate and was equilibrated against a reservoir consisting of 4 *M* sodium formate. The scale bar represents 0.1 mm.

SeMet SHARPIN crystals were obtained using the same two-dimensional grid procedure (with 4 *M* sodium formate as reservoir solution) and seeding the SeMet protein with the native seeds (Fig. 3 ▶).

### 2.3. Data collection

**Figure 4:**
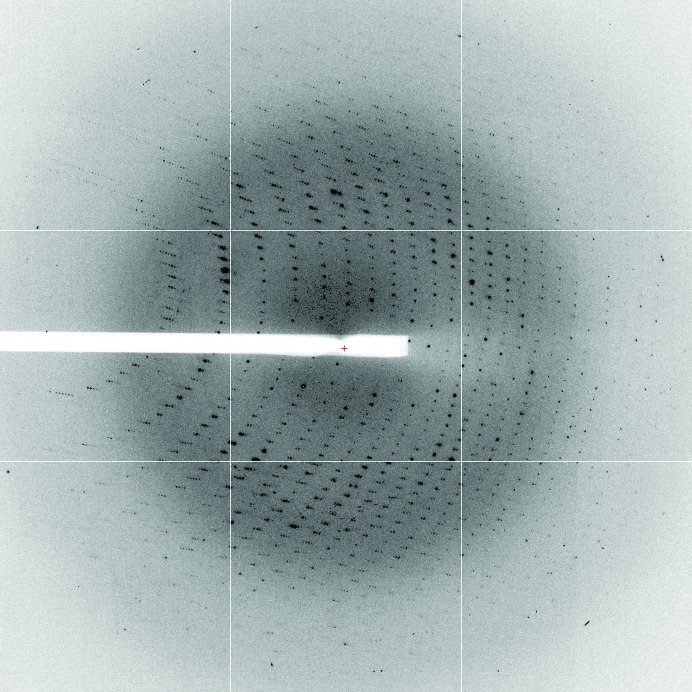
Representative X-ray diffraction image of a SeMet-SHARPIN crystal collected on the I04 beamline at the Diamond Light Source (Didcot, England). The crystal diffracted to 2.0 Å resolution (crystal-to-detector distance 289.53 mm).

Crystals were harvested from the drop with a cryoloop (Hampton Research) and flash-cooled in liquid nitrogen using 4 *M* sodium formate supplemented with 10%(*v*/*v*) glycerol as a cryoprotectant. A native data set was collected to a resolution of 2.6 Å at 100 K using a Rigaku MicroMax-007 HF with an R-AXIS IV detector. In order to solve the phase problem, a second data set was collected from a SeMet SHARPIN crystal at 100 K on beamline I04 at the Diamond Light Source (Didcot, England) at a wavelength of 0.9799 Å using an ADSC Q315r detector. A redundant data set of 90 frames with an oscillation range of 1° was collected (Fig. 4 ▶). The diffraction limit of the crystal was 2.0 Å. Data were indexed, integrated and scaled using the *HKL*-2000 package (Otwinowski & Minor, 1997 ▶). Systematic absences revealed that the crystals of SHARPIN 1–127 belonged to the primitive tetragonal space group *P*4_3_2_1_2 or *P*4_1_2_1_2, with unit-cell parameters *a* = *b* = 61.55, *c* = 222.81 Å (Table 1 ▶).

## 3. Results and discussion

There are four molecules per asymmetric unit, corresponding to a Matthews coefficient *V*_M_ of 2.03 Å^3^ Da^−1^ and a solvent content of 39.57% (Matthews, 1968 ▶). Structure determination by single-wavelength anomalous dispersion analysis has been described by Stieglitz *et al.* (2012 ▶). The structure factors and coordinates have been deposited in the Protein Data Bank (PDB entry 4emo).

Crystals appeared rapidly (within a day) using the two-dimensional grid method and were used for native data collection after 4 d. A control experiment was set up using 4 *M* sodium formate instead of microseed solution. No crystals grew, indicating that the introduction of seeds caused nucleation rather than the chemical bias resulting from the addition of mother liquor to the drop (St John *et al.*, 2008 ▶). It seems that an element of serendipity was involved in the nucleation of the original crystals at the site of the air bubble. No other heterogeneous nucleant, such as a piece of dust, was observed on the surface of the bubble. The use of these crystals as seeds in the two-dimensional grid screen increased the reproducibility of crystal growth.

Cross-seeding SeMet SHARPIN with native seeds (Stura & Wilson, 1992 ▶) using the two-dimensional grid screen resulted in the rapid growth of diffracting SeMet SHARPIN crystals in a one-step procedure.

The two-dimensional grid optimization strategy presented here has proved effective for the reproducible growth of diffracting native and SeMet SHARPIN crystals.

## Figures and Tables

**Table 1:** Data-collection statistics Values in parentheses are for the highest resolution shell.

	Native	SeMet
Source	In-house	Diamond I04
Wavelength (Å)	1.5418	0.9799
Resolution (Å)	30–2.6 (2.69–2.60)	30–2.0 (2.09–2.00)
Space group	*P*4_3_2_1_2/*P*4_1_2_1_2	*P*4_3_2_1_2/*P*4_1_2_1_2
Unit-cell parameters (Å)	*a* = *b* = 61.396, *c* = 222.431	*a* = *b* = 61.55, *c* = 222.81
*V*_M_ (Å^3^ Da^−1^)	2.02	2.03
Total measurements	169348	213897
Unique reflections	13876	55100†
Average multiplicity	12.2 (11.5)	3.9 (3.8)
〈*I*/σ(*I*)〉	17.9 (2.7)	17.2 (2.9)
Completeness (%)	99.0	99.1
Wilson *B* factor (Å^2^)	59.8	39.1
*R*_merge_ ‡ (%)	9.8 (62.0)	7.1 (48.3)

† Friedel pairs are treated as separate reflections.

‡ *R*_merge_ = 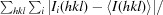 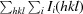, where 〈*I*(*hkl*)〉 is the average intensity of multiple *I_i_*(*hkl*) observations of symmetry-related reflections.
